# Elevated neutrophil to lymphocyte ratio and ischemic stroke risk in generally healthy adults

**DOI:** 10.1371/journal.pone.0183706

**Published:** 2017-08-22

**Authors:** Beomseok Suh, Dong Wook Shin, Hyung-Min Kwon, Jae Moon Yun, Hyung-Kook Yang, Eunmi Ahn, Hyejin Lee, Jin Ho Park, BeLong Cho

**Affiliations:** 1 Department of Family Medicine & Health Promotion Center, Seoul National University Hospital, Seoul, Korea; 2 Department of Family Medicine & Supportive Care Center, Samsung Medical Center, Seoul, Korea; 3 Department of Neurology, Seoul Metropolitan Government-Seoul National University Boramae Medical Center, Seoul, Korea; 4 Cancer Survivorship Branch, National Cancer Control Institute, National Cancer Center, Goyang, Korea; 5 Hospice and Palliative Care Branch, National Cancer Control Institute, National Cancer Center, Goyang, Korea; Massachusetts General Hospital, UNITED STATES

## Abstract

Elevated neutrophil to lymphocyte ratio (NLR) has been reported as a marker for chronic inflammation, associated with poor prognosis in ischemic stroke patients, but there has been no study that investigated its association with ischemic stroke risk. This study was conducted to investigate elevated NLR as an independent risk factor for ischemic stroke incidence. Our retrospective cohort study included 24,708 generally healthy subjects aged 30–75 who received self-referred health screening at Seoul National University Hospital. Data on ischemic stroke incidence was retrieved from national medical claims registry. Median follow-up time was 5.9 years (interquartile range 4.2 years). Adjusted for major cardiovascular risk factors, compared to subjects with NLR<1.5, subjects with 2.5≤NLR<3.0, 3.0≤NLR<3.5, and NLR≥3.5 had elevated risk for ischemic stroke incidence with aHR (95% CI) of 1.76 (1.09–2.84), 2.21 (1.21–4.04), and 2.96 (1.57–5.58), respectively. NLR showed significant improvement in discrimination for ischemic stroke incidence compared to traditional cardiovascular risk factors (C-index 0.748 vs. 0.739, P = 0.025). There was significant net improvement in reclassification in Framingham risk for ischemic stroke incidence after addition of NLR, with IDI 0.0035 (P<0.0001), and NRI 6.02% (P = 0.0015). This reclassification for ischemic stroke incidence by NLR was markedly pronounced among subjects with atrial fibrillation with CHA_2_DS_2_-VASc<2 (NRI 42.41%, P = 0.056). Our study suggests elevated NLR to be an independent risk factor for ischemic stroke incidence in generally healthy adults. Future studies are needed to validate our results and further assess how subjects with elevated NLR should be managed within current guidelines.

## Introduction

Cardiovascular disease (CVD) remains to be a leading cause of death and major public health burden with high prevalence [1]. Conventional risk prediction models for CVD play a major role in primary prevention measures, but these models often fail to identify a large proportion of subjects who eventually develop CVD [2]. Implementation of novel nontraditional risk factors which add predictive value for CVD is needed to improve prevention and management of CVD.

The association between chronic inflammation and atherosclerosis has been well established as the main model of pathogenesis for CVD [3]. For instance, elevated C-reactive protein (CRP), a well-studied marker for chronic inflammation, has been shown to be an independent risk factor for both coronary heart disease (CHD)[4] and ischemic stroke events [5], and has been suggested to be implemented in risk prediction models [6].

Neutrophil to lymphocyte ratio (NLR) is another marker for inflammation that has been reported to be associated with CHD incidence in asymptomatic subjects [7], and poor prognosis in patients with acute coronary syndromes [8]. Although NLR has been associated with poor short-term outcome among ischemic stroke patients [9], and ischemic stroke among patients with non-valvular atrial fibrillation (AF) [10], there has been no study on the association of NLR with future ischemic stroke incidence among generally healthy subjects. We have therefore investigated elevated NLR as a risk factor for ischemic stroke incidence in a generally healthy population.

## Methods

### Study population

Our study population was based on 28,075 Korean subjects from age 30 to 75 who received general health screening at Seoul National University Hospital (SNUH) consecutively during 2003–2010. As the study population comprised of consecutively sampled retrospective data, no formal calculation of sample size was done. Subjects with history of cancer (*N* = 214), CHD (*N* = 348), ischemic stroke (*N* = 142), congestive heart failure (*N* = 82), valvular heart disease (*N* = 29), peripheral vascular disease (*N* = 317), chronic obstructive lung disease (*N* = 88), complicated diabetes (with end-organ damage) (*N* = 128), end-stage renal disease (with estimated glomerular filtration rate (MDRD) <30 ml/min) (*N* = 17), major rheumatic diseases, including rheumatic arthritis, and systemic lupus erythematosis (*N* = 238), and those with missing laboratory results or insufficient medical records (*N* = 1543) were excluded, in respect to the first visitation. Additionally, subjects with serum white blood cell count (WBC) greater than 12.0 x10^9^/L (*N* = 221) were excluded to exclude subjects with probable acute inflammation at baseline. As a result, a total of 24,708 Korean adults aged 30–75 were enrolled. The SNUH Institutional Review Board approved our study and the requirement for informed consent was waived.

### Data collection

Self-reported questionnaire and physician interview was implemented to retrieve medical history information. Serum total cholesterol (TC), low-density lipoprotein (LDL), high-density lipoprotein (HDL), hemoglobin A1c (HbA1c), creatinine, and WBC with differential were measured after a minimum 12-hour fasting period. WBC counts were quantified using an automated blood cell counter (ADVIA 2120, Bayer, NY, USA). Blood pressure (BP), weight, and height were measured using a standardized protocol. Body mass index (BMI) was categorized according to the WHO Asia-Pacific criteria. Hypertension was defined as previous diagnosis of hypertension and/or use of hypertension medication or BP≥140/90 mmHg. Diabetes was defined as previous diagnosis of diabetes and/or use diabetes medication or HbA1c≥6.5%. Hyperlipidemia was defined previous diagnosis of hyperlipidemia and/or use of lipid lowering agents or LDL≥160 mg/dl or TC≥240 mg/dl.

We used data from the Korean National Health Insurance medical service claims registry that covers almost all Korean citizens (estimated to be around 97%). Diagnosis registry based on International Classification of Diseases, 10^th^ revision (ICD-10) was used. History of cancer (C00-C97), CHD (I20-I25), ischemic stroke (I63), congestive heart failure (I50), valvular heart disease (I34-I37), peripheral vascular disease (I70, I73-I74), chronic obstructive lung disease (J42-J44), complicated diabetes mellitus (G59.0, G63.2. H28.0, M14.2, N08.3), major rheumatic diseases (M05, M32-M36), and AF (I48) was defined when the respective diagnosis codes were registered within 1 year from the first visitation date. Ischemic stroke incidence (as outcome variable) was defined for cases where the claims data for ischemic stroke was registered after the date of visitation. Ischemic stroke incidence was followed-up until December 31, 2011 with median follow-up of 5.9 years (interquartile range 4.2). As subjects with history of CHD, ischemic stroke, congestive heart failure, valvular heart disease, and peripheral vascular disease were excluded for the study population, CHA_2_DS_2_-VASc scores[11] were determined using only age, sex, hypertension, and diabetes.

### Statistical analysis

NLR was defined as neutrophil count divided by lymphocyte count, as previously described. Subjects were categorized by their NLR levels: NLR<1.5 (median), 1.5≤NLR<2.0 (75th), 2.0≤NLR<2.5 (90th), 2.5≤NLR<3.0 (95th), 3.0≤NLR<3.5 (99th percentile), and NLR≥3.5.

Descriptive statistics was used to determine the basic characteristics of the study population. The association of elevated NLR and ischemic stroke incidence was evaluated by using Cox proportional hazards regression analysis, adjusted for age, sex, smoking, systolic BP, HbA1c, TC, HDL, medication for hypertension, and diabetes. Ischemic stroke incidence was compared for short-term follow-up (within 5 years) with long-term follow-up (beyond 5 years). Kaplan-Meier curves were shown to describe the cumulative risk of ischemic stroke by NLR levels. Incremental predictive value by improvement in discrimination by NLR for ischemic stroke was determined by comparing Harrell’s C-index of different Cox proportional hazards regression models with or without NLR included. Also, integrative discriminative improvement (IDI) and net reclassification improvement (NRI) for ischemic stroke by NLR was calculated to evaluate reclassification by NLR [7]. For NRI, risk categorization cutoff values of 5%, and 10% was used to stratify subjects with low, intermediate, and high risk, in order to avoid extrapolation beyond the range of our data, and also taking into account the relatively short follow-up period (median follow-up of 5.9 years) of our study, as similarly implemented previously [12]. Reclassification for ischemic stroke risk by NLR was also separately evaluated for subjects with AF, for all subjects with AF at baseline (*N* = 383) and for those with CHA_2_DS_2_-VASc<2 (*N* = 236). STATA version 12.1 (StataCorp, College Station, Texas, USA) was used to statistical analyses. Statistical significance was defined based on P*<*0.05.

## Results

### Characteristics of study population

The mean age of the study population was 51.8 ± 10.4 years. Males comprised of 49.9% of the study population, 18.3% were current smokers, and 34.0% were obese, as shown in Table 1. Subjects with AF comprised of 1.6% of study population.

**Table 1:** Characteristics of study population.

	NLR<1.5	1.5≤NLR<2.0	2.0≤NLR<2.5	2.5≤NLR<3.0	3.0≤NLR<3.5	NLR≥3.5	Total
**N, No.(%)**	12054 (48.8)	7073 (28.6)	3266 (13.2)	1356 (5.5)	592 (2.4)	367 (1.5)	24708
**Age, No. (%), year**							
30–39	1587 (13.2)	999 (14.1)	461 (14.1)	179 (13.2)	90 (15.2)	41 (11.2)	3357 (13.6)
40–49	3335 (27.7)	2125 (30.1)	973 (29.8)	439 (32.3)	155 (26.2)	97 (26.4)	7124 (28.8)
50–59	4319 (35.8)	2230 (31.5)	958 (29.3)	382 (28.2)	155 (26.2)	113 (30.8)	8157 (33.0)
60–69	2413 (20.0)	1405 (19.9)	686 (21.0)	283 (20.9)	150 (25.3)	88 (24.0)	5025 (20.4)
70–79	400 (3.3)	314 (4.4)	188 (5.8)	73 (5.4)	42 (7.1)	28 (7.6)	1045 (4.2)
**Male sex, No. (%)**	5986 (49.7)	3495 (49.4)	1655 (50.7)	689 (50.8)	294 (49.7)	206 (56.1)	12325 (49.9)
**Smoking, No. (%)**	2063 (17.1)	1330 (18.8)	674 (20.6)	276 (20.4)	98 (16.6)	73 (19.9)	4514 (18.3)
**Body mass index, No. (%), kg/m**^**2**^							
<20	957 (7.9)	626 (8.8)	336 (10.3)	154 (11.3)	75 (12.7)	39 (10.6)	2187 (8.9)
20–22.9	3463 (28.7)	2081 (29.4)	975 (29.8)	443 (32.7)	192 (32.4)	137 (37.3)	7291 (29.5)
23–24.9	3345 (27.8)	1945 (27.5)	913 (28.0)	371 (27.4)	152 (25.7)	104 (28.3)	6830 (27.6)
25–29.9	3920 (32.5)	2177 (30.8)	950 (29.1)	354 (26.1)	158 (26.7)	78 (21.3)	7637 (30.9)
≥30	369 (3.1)	244 (3.5)	92 (2.8)	34 (2.5)	15 (2.5)	9 (2.5)	763 (3.1)
**HTN, No. (%)**	2586 (21.5)	1548 (21.9)	771 (23.6)	307 (22.6)	153 (25.8)	90 (24.5)	5455 (22.1)
**DM, No. (%)**	2071 (17.2)	1275 (18.0)	617 (18.9)	263 (19.4)	121 (20.4)	79 (21.5)	4426 (17.9)
**HL, No. (%)**	3400 (28.2)	1867 (26.4)	849 (26.0)	304 (22.4)	133 (22.5)	89 (24.3)	6642 (26.9)
**AF, No. (%)**	161 (1.3)	108 (1.5)	63 (1.9)	35 (2.6)	9 (1.5)	7 (1.9)	383 (1.6)

Abbreviations: HTN, hypertension; DM, diabetes mellitus; HL, hyperlipidemia; AF, atrial fibrillation

### Association of elevated NLR with ischemic stroke incidence

**Fig 1:**
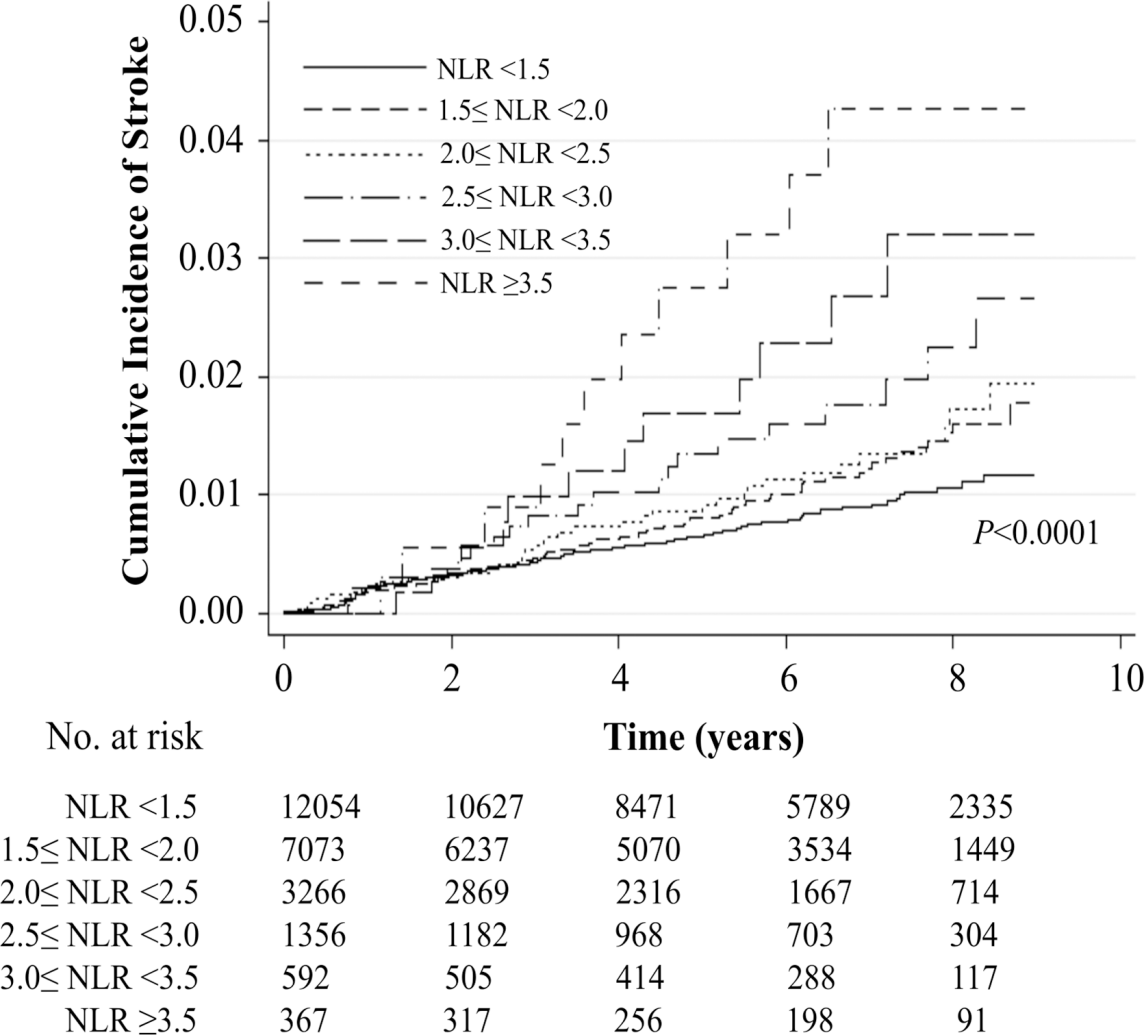
Kaplan-Meier curves for ischemic stroke incidence by neutrophil to lymphocyte ratio levels. Compared to subjects with NLR<1.5, subjects with 2.5≤NLR<3.0, 3.0≤NLR<3.5, and NLR≥3.5 had elevated risk for ischemic stroke incidence with aHR (95% CI) of 1.76 (1.09–2.84), 2.21 (1.21–4.04), and 2.96 (1.57–5.58), respectively, adjusted for major cardiovascular risk factors. Log-rank test showed P<0.0001.

Adjusted for major cardiovascular risk factors, compared to subjects with NLR<1.5, subjects with 2.5≤NLR<3.0, 3.0≤NLR<3.5, and NLR≥3.5 had elevated risk for ischemic stroke incidence with aHR (95% CI) of 1.76 (1.09–2.84), 2.21 (1.21–4.04), and 2.96 (1.57–5.58), respectively, with P-for-trend <0.001 (Fig 1, Table 2). Ischemic stroke incidence was shown to be generally similar for events within 5 years of follow-up and for events beyond 5 years of follow-up (Table 2).

**Table 2:** Neutrophil to lymphocyte ratio and ischemic stroke incidence.

	Person-year	Event N	Rate*	HR (95% CI)	aHR (95% CI)†
*For all events*					
NLR<1.5	66343.4	91	1.37	1	1
1.5≤NLR<2.0	39537.5	71	1.80	1.31 (0.96–1.78)	1.23 (0.90–1.68)
2.0≤NLR<2.5	18229.5	36	1.97	1.44 (0.98–2.11)	1.29 (0.87–1.90)
2.5≤NLR<3.0	7594.4	21	2.77	2.01 (1.25–3.23)	1.76 (1.09–2.84)
3.0≤NLR<3.5	3251.1	12	3.69	2.69 (1.47–4.91)	2.21 (1.21–4.04)
NLR≥3.5	2063.2	11	5.33	3.86 (2.06–7.21)	2.96 (1.57–5.58)
*For events within 5 years*					
NLR<1.5	14471.9	68	4.70	1	1
1.5≤NLR<2.0	7917.0	48	6.06	1.29 (0.89–1.86)	1.22 (0.84–1.77)
2.0≤NLR<2.5	3670.6	25	6.81	1.40 (0.88–2.23)	1.12 (0.70–1.80)
2.5≤NLR<3.0	1426.7	15	10.51	2.27 (1.30–3.98)	1.86 (1.06–3.27)
3.0≤NLR<3.5	610.2	8	13.11	2.86 (1.37–5.95)	1.97 (0.93–4.15)
NLR≥3.5	413.6	8	19.34	4.12 (1.98–8.58)	3.01 (1.42–6.41)
*For events beyond 5 years*					
NLR<1.5	51871.5	23	0.44	1	1
1.5≤NLR<2.0	31620.5	23	0.73	1.63 (0.92–2.91)	1.53 (0.85–2.72)
2.0≤NLR<2.5	14554.4	11	0.76	1.66 (0.81–3.41)	1.64 (0.80–3.38)
2.5≤NLR<3.0	6178.4	6	0.97	2.14 (0.87–5.25)	1.97 (0.80–4.85)
3.0≤NLR<3.5	2640.8	4	1.51	3.44 (1.19–9.96)	2.96 (1.02–8.63)
NLR≥3.5	1650.8	3	1.82	3.79 (1.14–12.63)	2.93 (0.88–9.99)

*Rate per 1,000 person-year

†Adjusted for age, sex, smoking, systolic blood pressure, total cholesterol, high-density lipoprotein, hemoglobin A1c, medication for hypertension, and medication for diabetes.

Abbreviations: NLR, neutrophil to lymphocyte ratio

### Incremental predictive value of NLR for ischemic stroke incidence

Predictive value for ischemic stroke incidence of the regression model with NLR added was shown to have significant incremental predictive value with C-index 0.776 (95% CI 0.746–0.807) compared to the basic model with traditional cardiovascular risk factors (C-index 0.739, 95% CI 0.708–0.770, P<0.001), as shown in Table 3. This addition of NLR also resulted in significant reclassification of 6.02% (95% CI 2.31–9.73, P = 0.0015) of overall subjects (Tables 3 & 4), with IDI 0.0035 (95% CI 0.0018–0.0054, P<0.0001). This was comparable to the addition of AF, to the basic model with traditional cardiovascular risk factors that resulted in 8.31% (95% CI 4.34–12.28, P<0.0001) reclassification for ischemic stroke incidence.

**Table 3:** Incremental predictive value of neutrophil to lymphocyte ratio for ischemic stroke incidence.

	Base model*	Base model with NLR	Base model with AF
*Discrimination*			
Harrell's C-index (95% CI)	0.739 (0.708–0.770)	0.748 (0.718–0.779)	0.776 (0.746–0.807)
P-value (vs. base model)	-	0.025	<0.001
*Reclassification*			
IDI (95% CI)	-	0.0035 (0.0018–0.0054)	0.0087 (0.0040–0.0134)
P-value	-	<0.0001	0.0003
NRI (%) (95% CI)	-	6.02 (2.31–9.73)	8.31 (4.34–12.28)
P-value	-	0.0015	<0.0001

* Model with age, sex, smoking, systolic blood pressure, total cholesterol, high-density lipoprotein, hemoglobin A1c, medication for hypertension, and medication for diabetes.

Abbreviations: NLR, neutrophil to lymphocyte ratio; AF, atrial fibrillation; IDI, integrated discrimination improvement; NRI, net reclassification improvement.

**Table 4:** Reclassification table for ischemic stroke incidence by neutrophil to lymphocyte ratio.

Base model*	Base model with NLR				Risk reclassification	
*Event (+)*	<5%	5–10%	≥10%	Total	Lower	Higher
<5%	197	11	0	208	NA	11
5–10%	1	21	7	29	1	7
≥10%	0	2	3	5	2	NA
Total	198	34	10	242		
*Event (-)*						
<5%	23,893	129		24,022	NA	129
5–10%	92	275	22	389	92	22
≥10%	0	16	39	55	16	NA
Total	23,985	420	61	24,466		

* Model with age, sex, smoking, systolic blood pressure, total cholesterol, high-density lipoprotein, hemoglobin A1c, medication for hypertension, and medication for diabetes.

Abbreviations: NLR, neutrophil to lymphocyte ratio; NA, not applicable

### Incremental predictive value of NLR for ischemic stroke incidence among subjects with atrial fibrillation

The addition of NLR to the model adjusted for traditional cardiovascular risk factors resulted in NRI 19.62% (95% CI -18.85–58.09, P = 0.158) among subjects with baseline AF (Table 5). This was even more marked among AF patients with CHA_2_DS_2_-VASc<2 with NRI 42.41% (95% CI -9.87–94.70, P = 0.056), although both results lacked statistical power, most likely due to insufficient number of subjects.

**Table 5:** Reclassification of subjects with atrial fibrillation for ischemic stroke incidence by neutrophil to lymphocyte ratio.

	Subjects with AF (All)	Subjects with AF (CHA_2_DS_2_-VASc<2)
N	383	236
Event N	28	15
IDI (95% CI)	0.0122 (-0.0103–0.0347)	0.0335 (-0.0112–0.0782)
P-value	0.144	0.062
NRI (%) (95% CI)	19.62 (-18.85–58.09)	42.41 (-9.87–94.70)
P-value	0.158	0.056

Abbreviations: AF, atrial fibrillation; IDI, integrated discrimination improvement; NRI, net reclassification improvement.

## Discussion

In this relatively large-scale retrospective cohort study on generally healthy screened population, subjects with elevated NLR were demonstrated to have elevated risk for ischemic stroke incidence. NLR was also shown to add incremental predictive value in predicting ischemic stroke incidence to traditional cardiovascular risk factors. Clear dose-response relationship by NLR levels adds plausibility to our results. Pre-existing conditions have been extensively excluded for accurate assessment of the relationship between NLR and ischemic stroke risk. The readily available nature of NLR, which is commonly included in routine blood tests, adds value to our findings.

The association between chronic inflammation and CVD has been extensively studied [13]. Elevated WBC was shown to be associated with CVD [8], where among the different leukocyte types, neutrophils provided the highest predictive value, consistent with other studies that have revealed neutrophils to contribute significantly to pathogenesis of atherosclerotic diseases, contrary to previous consensus [14]. Interestingly, NLR has been reported to have the highest predictive value compared to individual cell counts [8], and recently, a study reported increased prospective CHD mortality among asymptomatic patients with elevated NLR [7]. Although there has been no previous prospective study on the association between NLR and ischemic stroke, many have reported their cross-sectional association [9, 10]. Consistent with such previous studies, the results of our study (to report NLR to be associated with ischemic stroke risk) are hardly surprising.

What is more important is whether NLR has value in being included in risk prediction models. Our results consistently show NLR does add incremental predictive value to traditional cardiovascular risk factors in predicting ischemic stroke risk. In a previous study, NLR has been shown to be only weakly correlated (correlation coefficient = 0.11, P<0.001) to CRP, and NLR was shown to be predictive of CHD mortality independent of CRP [7]. Although elevated CRP was shown to have similar relative risk for ischemic stroke compared to that for CHD in a large-scale meta-analysis [15], it was shown to add insignificant degree of predictive value for ischemic stroke in terms of both discrimination and reclassification in another subsequent large-scale meta-analysis [6]. In comparison (although a direct quantitative comparison cannot be made), our results show elevated NLR to provide significant increase in C-index by 0.009, and considerably large NRI of 6.02% for ischemic stroke incidence. These results are notable because they suggest NLR may be used in prediction models for ischemic stroke, and it may provide additional information to what is available with traditional risk factors. Our results are very comparable to a previous study that reported elevated NLR to have NRI of 6.6% for predicting CHD mortality [7], and another that reported elevated neutrophil count to have NRI of 5.0% for predicting CVD mortality [16].

Interestingly, in this study, it was shown that neutrophil count is a stronger predictor of CVD mortality compared to CRP [16]. This was consistent in our study where NLR was shown to have possibly the highest incremental predictive value for ischemic stroke incidence compared to other inflammatory markers including WBC, CRP, and ESR, as shown in S1 Table, although our results remain inconclusive due to the indirect nature of the comparison and the limited number of subjects who received laboratory tests for CRP and/or ESR. This tentatively suggests that NLR may go beyond being a collinear counterpart of CRP, reflecting different biological aspects of inflammation that may have higher predictive value for ischemic stroke. Future studies are needed to determine whether NLR should be included in current guidelines for CVD risk assessment, similar to how CRP is recommended in intermediate or borderline risk subjects [17]. It is to be noted if NLR has similar predictive value for CVD compared to CRP, NLR would be the preferable test due to its availability, included in routine lab tests.

Assessment of future ischemic stroke risk, rather than cardiovascular risk in general, is especially clinically important for patients with AF, where appropriate thromboprophylaxis is central to the management of AF [11]. Various cardiac, renal function, coagulation, and inflammatory markers are being studied and some have shown improved risk stratification for ischemic stroke in AF patients.[18] However, there is lack of evidence to suggest CRP provides incremental predictive value for ischemic stroke incidence, although there has been no study that investigated reclassification improvement for ischemic stroke incidence by CRP in AF patients [18]. According to our results, a tendency for considerable improvement in reclassification by NLR for ischemic stroke incidence in subjects with AF was observed (Table 3), although there was insufficient statistical significance, likely due to the small number of subjects. This needs to be investigated in future studies with a large population of AF subjects. NLR may play a significant role in ischemic stroke risk management among AF patients.

While it is widely accepted that chronic inflammation is a central component of CVD, there is little evidence elevated CRP is a direct cause. Although studies have shown decrease in CRP levels in statin therapy to be associated with better cardiovascular outcomes regardless of LDL cholesterol levels [19], genetic studies have failed to show CRP to have direct effect on CVD [20]. Similar investigations are needed to determine whether elevated NLR is a direct cause for CVD pathogenesis. As statin therapy has been shown to inhibit proinflammatory effects of neutrophils [21], the possibility that activated neutrophils are a direct cause for CVD is viable. Inhibition of neutrophil function may be a promising target for development of drugs that may serve as CVD prevention measures. Our study suggests this may be specifically relevant for ischemic stroke prevention.

In addition to chronic inflammation, there may be other pathophysiological explanations for the association of increased NLR and ischemic stroke. For example, neutrophil extracellular trap formation with increased neutrophil activity has been associated with thrombosis [22]. This is a biologically plausible explanation to the relationship between increased NLR and ischemic stroke.

Our study has several limitations that need to be addressed. First, incidence data in our study were retrieved from national medical claims registry that is susceptible to inaccuracy. In addition, clinical characteristics of stroke events were not available, which may have provided meaningful insights. However, there is currently no reason to believe there is systematic difference of such information between subjects with different levels of NLR. Second, despite our study population being based on a screened population of generally healthy subjects, our results cannot be generalized, because it was not a population-based study. Our results need to be validated by large-scale population-based prospective cohort studies.

## Conclusion

Nonetheless, the results of our study have the potential to have important clinical implications. To our knowledge, this study is the first to show that elevated NLR may be an independent risk factor for ischemic stroke incidence, possibly used as a clinical indicator for ischemic stroke. Future studies are needed to replicate our results and specifically investigate whether it would be beneficial to include NLR in prediction models for ischemic stroke, and how subjects with elevated NLR should be managed in terms of ischemic stroke prevention.

## Supporting information

S1 Table Comparison of various inflammatory markers in predictive value for ischemic stroke incidence. Each model includes age, sex, smoking, systolic blood pressure, total cholesterol, high-density lipoprotein, hemoglobin A1c, medication for hypertension, and medication for diabetes in addition to each of the corresponding inflammatory marker.
Abbreviations: NLR, neutrophil to lymphocyte ratio; WBC, white blood cell; CRP, C-reactive protein; ESR, erythrocyte sedimentation rate; IDI, integrated discrimination improvement; NRI, net reclassification improvement.
(DOC)
