# Supplementary material for: Elevated neutrophil to lymphocyte ratio and ischemic stroke risk in generally healthy adults
Source: PLoS One. 2017 Aug 22;12(8):e0183706. doi: 10.1371/journal.pone.0183706 (PMC5567907; doi:10.1371/journal.pone.0183706)
Supplement: S1 Table — Each model includes age, sex, smoking, systolic blood pressure, total cholesterol, high-density lipoprotein, hemoglobin A1c, medication for hypertension, and medication for diabetes in addition to each of the corresponding inflammatory marker. Abbreviations: NLR, neutrophil to lymphocyte ratio; WBC, white blood cell; CRP, C-reactive protein; ESR, erythrocyte sedimentation rate; IDI, integrated discrimination improvement; NRI, net reclassification improvement. (DOC) [file pone.0183706.s001.doc]

| **S1 Table . Comparison of Various Inflammatory Markers in Predictive Value for Ischemic Stroke Incidence** | | | | |
| --- | --- | --- | --- | --- |
|  | NLR Model | WBC Model | CRP Model | ESR Model |
| N | 24,708 | 24,708 | 8,767 | 7,202 |
| IDI (95% CI) | 0.0035 (0.0018-0.0054) | 0.0005 (0.0001-0.0009) | 0.0003 (0.0001-0.0005) | 0.0001 (-0.0001-0.0003) |
| P-value | <0.0001 | 0.0790 | 0.0443 | 0.3402 |
| NRI (%) (95% CI) | 6.02 (2.31-9.73) | 1.15 (-0.99-3.30) | 3.37 (-0.44-7.19) | 1.38 (-1.38-4.14) |
| P-value | 0.0015 | 0.2916 | 0.0833 | 0.3275 |
| Each model includes age, sex, smoking, systolic blood pressure, total cholesterol, high-density lipoprotein, hemoglobin A1c, medication for hypertension, and medication for diabetes in addition to each of the corresponding inflammatory marker.  Abbreviations: NLR, neutrophil to lymphocyte ratio; WBC, white blood cell; CRP, C-reactive protein; ESR, erythrocyte sedimentation rate; IDI, integrated discrimination improvement; NRI, net reclassification improvement. | | | | |
